# Proteomic and Membrane Lipid Correlates of Re-duced Host Defense Peptide Susceptibility in a *snoD* Mutant of *Staphylococcus aureus*

**DOI:** 10.3390/antibiotics8040169

**Published:** 2019-09-28

**Authors:** Christian Kohler, Richard A. Proctor, Arnold S. Bayer, Michael R. Yeaman, Michael Lalk, Susanne Engelmann, Nagendra N. Mishra

**Affiliations:** 1 Universität Greifswald, Institut für Mikrobiologie und Molekularbiologie,17487 Greifswald, Germany; 2 University of Wisconsin School of Medicine and Public Health, Madison, WI 53705, USA; rap@wisc.edu; 3 Division of Infectious Diseases, Los Angeles Biomedical Research Institute at Harbor-UCLA Medical Center Torrance, CA 90502, USAmryeaman@ucla.edu (M.R.Y.); 4 David Geffen School of Medicine at UCLA, Los Angeles, CA 90095, USA; 5 Division of Molecular Medicine, Los Angeles Biomedical Research Institute at Harbor-UCLA Medical Center, Torrance, CA 90502, USA; 6 University Greifswald, Institute of Biochemistry, 17487 Greifswald, Germany; lalk@uni-greifswald.de; 7 Institute for Microbiology, Technical University Braunschweig, Institute for Microbiology, 38106 Braunschweig, Germany; 8 Helmholtz Center for Infectious Research, Microbial Proteomics, 38124 Braunschweig, Germany

**Keywords:** *S. aureus*, snoD mutant, lipids, proteomics, tPMP resistance

## Abstract

We previously described a transposon mutant in *Staphylococcus aureus* strain SH1000 that exhibited reduced susceptibility to cationic thrombin-induced platelet microbicidal proteins (tPMPs). The transposon insertion site was mapped to the gene *snoD*, the staphylococcal *nuo* orthologue. Hence, further studies have been performed to understand how this mutation impacts susceptibility to tPMP, by comparing proteomics profiling and membrane lipid analyses of the parent vs. mutant strains. Surprisingly, the mutant showed differential regulation of only a single protein when cultivated aerobically (FadB), and only a small number of proteins under anaerobic growth conditions (AdhE, DapE, Ddh, Ald1, IlvA1, AgrA, Rot, SA2366, and SA2367). Corresponding to FadB impact on lipid remodeling, membrane fatty acid analyses showed that the *snoD* mutant contained more short chain anteiso-, but fewer short chain iso-branched chain fatty acids under both aerobic and anaerobic conditions vs. the parental strain. Based upon these proteomic and membrane compositional data, a hypothetical “network” model was developed to explain the impact of the *snoD* mutation upon tPMP susceptibility.

## 1. Introduction

Thrombin-induced platelet microbicidal proteins (tPMPs) represent an important component of innate immunity against endovascular infections, including those caused by both methicillin-susceptible and methicillin-resistant *Staphylococcus aureus* (MSSA; MRSA, respectively) [1]. For example, clinical *S. aureus* strains which exhibit reduced killing by low concentrations of tPMPs in vitro are associated with: i) endocarditis and vascular catheter-associated staphylococcemias (as compared to isolates from soft-tissue infections) [2]; ii) more prolonged bacteremia in patients with or without endocarditis [2,3]; and iii) increased persistence and/or reduced responsiveness to antibiotic treatment in humans [2] or experimental endocarditis [4,5,6]. Reduced susceptibility to tPMPs in vitro has also been correlated to a broad array of specific staphylococcal adaptive phenotypes: i) decreased membrane potential (Δψ) associated with interruption of either aerobic or anaerobic electron transport systems [7,8]; ii) increased surface positive charge due to enhanced lysinylation of phosphatidyl-glycerol (PG) and/or D-alanylation of wall teichoic acids [8]; iii) altered membrane order (i.e., extremes of fluidity or rigidity) [8,9,10]; iv) distinct changes in carotenoid content [9]; and v) alterations of the QacA membrane transporter [11]. 

Previously, we found that a transposon-induced mutant in the well-characterized MSSA strain, SH1000, was resistant to killing by low concentrations of tPMPs in vitro [4]. The transposon insert was mapped to a locus we termed *snoD*, for Staphylococcal *nuo* orthologue [12]. Phenotypically, in addition to reduced susceptibility to tPMPs, the mutation in *snoD* was associated with a reduced capacity to maintain Δψ over a standard growth cycle, as well as enhanced membrane fluidity [12]. 

Beyond *snoD*, previous attempts to delineate how specific genetic regulatory mechanism(s) and/or pathway(s) may be related to tPMP resistance have met with challenges. For example, our initial comparative gene expression profiles by microarray were not particularly revealing (unpublished), and suffered from several key limitations, especially: i) single growth phase time-point queries; and ii) aerobic growth conditions, given that the *snoD* operon might be most important in terms of anaerobic oxidative activities [12]. Therefore, to gain further insights into potential mechanism(s) behind the above phenotypes observed in the *snoD* mutant, we compared the proteomic and membrane fatty acid compositional profiles of this mutant with that of its isogenic parental strain, under aerobic as well as anaerobic conditions.

## 2. Methods

### 2.1. Bacterial Strains and Growth Conditions

The study strain-pair has been described in detail before [12]. In brief, MSSA parental strain, SH1000, and its isogenic *snoD* mutant were used in the present study. The *snoD* locus was insertionally inactivated by transposon mutagenesis [12], and the mutant designated as SH1000-98. Staphylococcal *snoD*, (like *mrp*D of *Bacillus subtilis*), is found in a seven-gene operon [13]. These genes encode homologous proteins, and the overall architecture of at least two operons is indistinguishable [13]. *snoD* encodes the unique gene product which is affected by the transposon mutation only [12]. The *sno* (mnh) operon was reported as Na+/H+ antiporter function before [14]. As previously published, the SH-1000 parental strain is more susceptible to killing by sub-physiologic concentrations of tPMPs in vitro as compared to the *snoD* mutant, SH1000-98 (21 ± 10% vs. 59 ± 2% survival after two hours exposure to 2 µg/mL tPMPs bioequivalent units, respectively; *p* < 0.05) [4]. This platelet peptide preparation has been previously shown to contain a mixture of tPMPs, wherein tPMP-1 is of quantitative predominance. In addition, the *snoD* mutant differs from the parental strain in the following parameters: i) cross-resistant to the membrane-active cationic peptide, protamine; and ii) enhanced membrane fluidity. 

The bioactivity of the tPMP preparations was performed as described previously [15]. For protein preparation and lipid membrane analyses, 100 mL tryptic soy broth (TSB) (Oxoid, Wesel, Germany) was inoculated with exponentially growing cells of an overnight culture to an initial optical density at 540 nm (OD_540_) of 0.05. Cells were cultivated under vigorous agitation at 37 °C in baffled 500 mL Erlenmeyer flasks to an OD_540_ of 0.7. Afterwards, 50 mL of the culture were shifted to anaerobic conditions by cultivating in 50 mL Falcon Tubes under gentle agitation at 37 °C. Anaerobic conditions were verified by using the redox indicator, resazurin, as previously described [16].

### 2.2. Proteomic Analyses

Cytoplasmic proteins of the parental strain and its isogenic *snoD* mutant were prepared in parallel from aerobically and anaerobically grown cells. Cells were harvested by centrifugation (4 °C, 10 min at 7000× *g*), and the resulting cell pellet was washed twice with ice-cold Tris-EDTA buffer. For cell lysis, cells were suspended in 1 mL (10 mM Tris, 1 mM EDTA, 1 mM phenylmethylsulfonyl fluoride, pH 7.5) and placed in screw-cap microtubes (Sarstedt, Germany) containing 500 μL of glass beads (diameter 0.10 to 0.11, Sartorius, Goettingen, Germany). Cells were mechanically disrupted as described [17]. The lysate was centrifuged twice at 21,000× *g* (4 °C) for 25 and 45 min, respectively. The protein concentration of the supernatant was determined using Roti-Nanoquant (Roth, Karlsruhe, Germany) and protein solutions were stored at −20 °C until analyzed. To resolve the cytoplasmic proteins, 2-dimensional polyacrylamide gel electrophoresis (2D PAGE) was performed as described [17,18]. The proteins separated by 2D PAGE were stained with colloidal Coomassie brilliant blue [19] and the gels were scanned with a light scanner with an integrated transparency unit (Quatographic, Braunschweig, Germany). The 2D gels were then analyzed with the software Delta2D (Decodon GmbH, Greifswald, Germany). To adjust for differences in the amount of cytoplasmic proteins detected on 2D gels of the parental vs. the mutant strain under aerobic, as well as anaerobic conditions, data sets obtained from three separate and independent replicates were used.

For identification of differentially expressed proteins, the respective spots were cut from the gels and digested with trypsin. The resulting peptides were analyzed by matrix-assisted laser desorption ionization-time of flight mass spectrometry (MALDI-TOF MS) as described [20,21] with several modifications detailed elsewhere [17]. The combined mass spectroscopy (MS) and MS/MS peak identity results were searched against the *S. aureus* 8325 protein data base with the Mascot search engine version 2.1.0.4. (Matrix Science; London, United Kingdom) using query parameters as published by Kohler et al. [17] where protein concentrations were considered different at *p* < 0.05.

### 2.3. Fatty Acid Extraction and Gas Chromatographic Analysis of Fatty Acid Methyl Esters (GC-FAME)

To determine the membrane fatty acid composition, colonies of SH1000 and SH1000-98 were freshly sub-cultured from original plates and grown on sheep blood agar in a quadrant pattern at 35 °C, in parallel aerobically and anaerobically (in BBL Gaspacks, producing an atmosphere of more than 15% CO_2_). A second subculture was streaked in order to fully acclimate the strains to these respective growth conditions. Approximately 20 mg of cell mass were then harvested after 24 hours from the late exponential phase of growth (3Q) and placed into 13 × 100 mm test tubes. Next, the cells were saponified, methylated, and fatty acid esters were extracted into hexane as described [9]. The resulting methyl ester mixtures were separated by an Agilent 5890 dual-tower gas chromatograph. Specifically, fatty acids were identified by a well-characterized microbial identification system, utilizing individual known fatty acids as positive controls (Sherlock 4.5; MIDI Inc.; Wilmington, DE). For statistical analysis, data were analyzed by the unpaired, two-tailed Student’s *t* test, with *p* < 0.05 representing a significant difference. A minimum of two independent runs were performed on different days.

### 2.4. Pathway Analysis and Bioinformatics

Gene functions were imputed based on known genomic relationships available through the University of Nebraska Transposon Mutant Library (formerly, NARSA) [22]. Pathway analysis and interpretive results were derived in part using information obtained from the Kegg database [23]. 

## 3. Results

### 3.1. Proteomics Analyses

Under aerobic conditions, changes in the proteome of the *snoD* mutant were surprisingly quite limited (Table 1). Only SA0224 (similar to FadB = 3-hydroxyacyl-CoA dehydrogenase) was increased in the *snoD* mutant. This enzyme is involved in the β-oxidation of fatty acids, as well as in the propanoate and butanoate pathways. In contrast, under anaerobic conditions, more significant changes in the proteome of the *snoD* mutant were observed. For example, the mutant showed increased Ddh, a lactic acid dehydrogenase, and Ald1, an amino acid dehydratase. Both of these enzymes oxidize NADH as they perform their enzymatic roles. Additionally, IlvA1, an enzyme involved in deamination of amino acids, was also increased. This enzyme intersects pathways involved in the formation of branched chain fatty acids. Moreover, DapE, which metabolizes lysine to N-succinyl-LL-2,6-diaminopimelate, was decreased in the *snoD* mutant relative to its parental counterpart under anaerobic conditions. The reduction in DapE would be expected to increase the pool of lysine available for lysinylation of the cell membrane, thereby increasing the relative positive charge of the cell surface. Earlier publications suggested that *snoD* might be in an operon concerned primarily with osmoprotection, and that the *snoD* mutant was less halotolerant than its parental strain [12]. Therefore, proteomic analyses were performed aerobically and anaerobically, in parallel, after growth of the strains under several salt concentrations (range of NaCl concentrations = 1 M, 2 M, 2.5 M, and 3 M). No significant differences in proteome profiles, aerobically or anaerobically, were found in the *snoD* vs. parental strains when challenged across this range of high salt concentrations (data not shown).

### 3.2. Fatty Acid Analyses

Bacterial growth entails a substantial portion of the acyl chains of lipids in membrane to maintain the optimum level of fluidity/rigidity [9,10,24]. Previous studies have shown that changes in the fatty acid composition of *S. aureus* can correlate with in vitro resistance to cationic host defense peptides, including tPMPs [9,10]. Hence, we examined fatty acid compositions of the parent and *snoD* mutant strains under both aerobic and anaerobic conditions. Among fatty acid species prevalent in *S. aureus*, large changes in membrane fluidity begin to occur at C17, and are even more pronounced at C15 and C13; hence, these species were grouped in this manner for our analyses. As can be seen in Table 2, both parental and mutant strains showed major fatty acid content differences anaerobically, with significantly decreased anteiso-branched chain, unsaturated-, and polyunsaturated-fatty acids. Also, the amount of short iso-branched fatty acids increased, while long iso-branched fatty acids decreased with a shift to anaerobic conditions. The *snoD* mutant contained more short chain anteiso-branched fatty acids and fewer short chain iso-branched fatty acids under both growth conditions as compared to the parental strain. In contrast, unsaturated fatty acid profiles were increased to an equivalent extent in both of the strains under anaerobic conditions (full fatty acid analysis shown as Table 3).

## 4. Discussion

**Figure 1:**
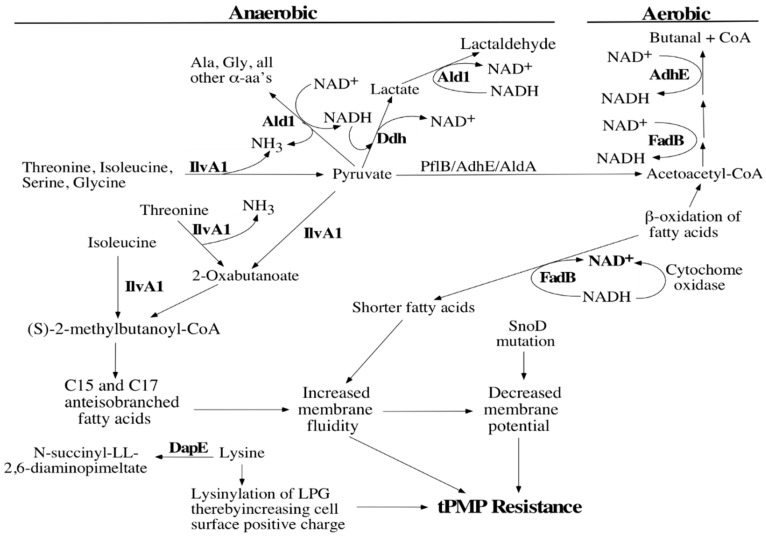
Integrative hypothetical model of reduced tPMP susceptibility in *S. aureus*. This hypothetical model was derived from bioinformatics analysis and incorporates the data presented in this paper and previous work. This model illustrates how *S. aureus* can adapt to a phenotype that has reduced susceptibility to tPMP with a single mutation in *snoD*. Abbreviations used in figure: AdhE = alcohol-acetaldehyde dehydrogenase; Ald1 = α-amino acid dehydrogenase; DapE = succinyl-diaminopimelate desuccinylase; Ddh = D-lactate dehydrogenase; FadB = 3-hydroxyacyl-CoA dehydrogenase; IlvA1 = Threonine/serine dehydrase; PflB = formate acetyltransferase; *snoD = S. aureus nuo* orthologue; tPMP = thrombin-induced platelet microbicidal protein.

The scope of significant proteomic changes in the *snoD* mutant strain was surprisingly limited, especially under aerobic conditions. In particular, only locus SA0224 (similar to FadB = 3-hydroxyacyl-CoA dehydrogenase) was differentially upregulated in the *snoD* mutant under aerobic conditions. FadB shortens fatty acid chains, which would be consistent with the previously documented increases in membrane fluidity in our *snoD* mutant [12]. In turn, this observation is consistent with prior linkage of increased membrane fluidity phenotypes with increased resistance of *S. aureus* to tPMPs in vitro [1,7,8,9,10,25]. Increased membrane fluidity is associated with proton leak and reduced membrane potential [12] with increased tPMP resistance, as shown in the model (Figure 1). As illustrated, β-oxidation of fatty acids by FadB yields NADH, which must then be oxidized for metabolism to continue, as a large number of dehydrogenases require this substrate as a co-factor. Because the cytochrome oxidase system would be fully functional aerobically, the products of central metabolism that generate NADH could be efficiently processed under aerobic conditions by cytochrome oxidase. Importantly, Sharma-Kuinkel et al. previously demonstrated that the *Fad* operon correlates with clonal complex type 30 (CC30) and may confer upon *S. aureus* the ability to adapt for survival in the face of cationic agents such as daptomycin [26]. In turn, the *Fad* operon may give staphylococcal strains virulence advantages in persistent or other human infections. 

More notable changes were identified in the proteome of the *snoD* mutant under anaerobic vs. anaerobic conditions, which is consistent with our previous findings, wherein differences in tPMP susceptibility were greatest under microaerophilic conditions [12]. While none of the proteins in the *sno* operon have NADH oxidase activity, these proteins may be linked to the upstream protein, Ndh2 oxidase, and the membrane potential-forming protein, MspA [27]. In any event, a consistent pattern is seen in the proteins induced under anaerobic conditions; i.e., these proteins are clearly linked to NADH oxidation. For example, the Ddh protein in *S. aureus* serves as a NAD^+^-dependent lactate dehydrogenase [28]. We found Ddh to be significantly upregulated in the *snoD* mutant under anaerobic conditions. Likewise, the Ald1 protein is over-represented anaerobically in the *snoD* mutant proteome as compared to its isogenic parental strain. Importantly, we previously reported that Ald1 expression aerobically was increased in the tPMP-resistant *hemB* and *menD* small colony variants, both of which have defects in oxidizing NADH [15,29]. 

As a unifying model, we have related the observed changes in Ddh and Ald1 profiles in the *snoD* mutant to the relative redox state, wherein pathways optimized to oxidize NADH anaerobically are likely operative (Figure 1). The relatively greater increase in AdhE in the parent probably relates to the fact that this protein may already be adequately expressed in the mutant, but needs to be induced in the parent to accomplish anaerobic oxidation of NADH. 

In addition to changes in NADH/NAD^+^ cycling in the *snoD* mutant, we identified protein perturbations that should facilitate changes in cell membrane lipid composition. In *S. aureus*, the IlvA1 protein acts as a threonine desaturase, which catalyzes the anaerobic formation of 2-oxobutanoate (α-ketobutyrate) and ammonia from threonine in a two-step reaction (Figure 1). IlvA1 shuttles amino acids towards (S)-2-methylbutanoyl-CoA, which would be expected to increase both the proportion of C15 and C17 anteiso-branched fatty acids and, consequently, the membrane fluidity. The ratio of anteiso- to short iso-branch chain lipids is approximately 2.6 in the *snoD* mutant, as compared to 2.1 in the parent. This shift in the lipid profile of the *snoD* mutant strain is similar to the membrane perturbations in *B. subtilis* during “cold adaptation” under anaerobic conditions [24], a modification that also increases membrane fluidity [30]. Anteiso-branched fatty acids produce more fluid membranes as compared to iso-branched chain fatty acids [31]); hence, the present results support the previously identified changes in membrane fluidity in the *snoD* mutant [10]. The “breakpoint” for changes in membrane fluidity occurs in iso-branched chain fatty acids at C15-to-C16 species. Thus, we used this breakpoint to group our data for relevant analyses. While the amount of anteiso-branched fatty acids is normally lower anaerobically (which would hypothetically render cell membranes more rigid), the anteiso-branched fatty acids in the *snoD* mutant are maintained at relatively higher levels, thereby allowing for greater fluidity than the parent. All of the changes in fatty acid length and branching favor increased membrane fluidity.

In theory, increased membrane fluidity may impact susceptibility to tPMPs and other cationic host defense peptides in at least two ways. First, the insertion capacity of these cationic peptides into target membranes is likely to be physically altered by such extremes of membrane order [1,7,8,9,10,25,32]. Second, a more fluid membrane allows for greater transmembrane leakage of protons, dissipating the Δψ [33], a phenotype that is critically involved in tPMP activity [7]. In direct support of this notion, the mutation in *snoD* is associated with a substantial decrease in Δψ [12]. One possible explanation for this observation is that increased fluidity would allow fewer tPMP molecules to insert into the cell membrane of the *snoD* mutant, limiting their membrane perturbing actions. This defect would also blunt their eventual penetration into the cytoplasmic compartment of the *snoD* mutant where these peptides are believed to exert multiple antimicrobial mechanisms [9,10,11,12,34]. Finally, reduced DapE content in the *snoD* mutant would decrease lysine breakdown (Figure 1). This would increase lysine availability for lysinylating phosphatidylglycerol (PG) and synthesizing this positively-charged membrane species, lysyl-PG; this event, in turn, would likely increase the relative positive surface charge in the *snoD* mutant, resulting in a “charge-repulsive milieu” against host cationic peptides, like tPMPs [8]. 

In summary, we have previously reported that mutation and loss-of-function of the *SnoD* complex in *S. aureus* is correlated with increased membrane fluidity and reduced Δψ, as well as with decreased NADH oxidation and increased tPMP resistance. This mutant appears to foster these adaptations by engaging alternative pathways to oxidize NADH, increase membrane fluidity, and redirect metabolism to allow for a relative increase in surface charge. Of most importance, by modifying the expression of only a very small group of proteins, the organism becomes more resistant to an important host defense peptide (tPMP), supporting its survivability under stress conditions.

## Figures and Tables

**Table 1:** Proteins of which the amount was affected by *snoD* mutation under anaerobic conditions.

Protein (Annotation From 315)	Function	Ratio log2 (SH1000/snoD)
**Increased in parent**		
AdhE (SA0143)	Acetaldehyde DH	1.46
(SA2367)	Theoretical protein	1.044
**Decreased in mutant**		
DapE (SA1814)	Lysine metabolism	1.021
(SA2366)	Theoretical protein	1.051
AgrA (SA1844)	Virulence regulator	1.287
**Increased in mutant**		
IlvA1 (SA1271)	Threonine/serine dehydrase	−4.17
Ald1 (SA1272)	α-amino acid dehydrogenase	−1.667
Ddh (SA2312)	D-lactate dehydrogenase	−1.006

**Table 2:** Summary fatty acid compositional differences between parent and *snoD* mutant.

Fatty Acid Species	SH1000-98		* *p* value	SH1000-WT		* *p* value
	Aerobic	Anaerobic		Aerobic	Anaerobic	
**Anteiso (C13–17)**	**64.49 ± 0.11**	39.07 ± 0.49	0.006	61.16 ± 1.16	35.85 ± 1.10	0.002
**Unsaturated Polyunsaturated subset (C20)**	3.58 ± 0.14 0.42 ± 0.01	12.81 ± 0.16 0.49 ± 0.01	0.0003 0.045	3.565 ± 0.40 0.315 ± 0.01	12.10 ± 0.09 0.42 ± 0.01	0.016 0.027
**Short Isos (C13–15)**	5.97 ± 0.08	14.69 ± 0.25	0.007	6.895 ± 0.13	16.60 ± 0.41	0.015
**Long Isos (C16–20)**	6.03 ± 0.05	5.205 ± 0.05	0.003	7.195 ± 0.08	5.30 ± 0.03	0.009

* *p*-value = aerobic vs. anaerobic.

**Table 3:** Complete fatty acid analysis of strain-pair under aerobic and anaerobic conditions.

Fatty Acid Species	Aerobic			Anaerobic		
	SH1000-WT	SH1000-98	*p*-value ^*^	SH1000-WT	SH1000-98	*p*-value ^**^
**13:0 iso**	-			0.325 ± 0.01	0.265 ± 0.02	
**13:0 anteiso**	-	0.105 ± 0.01		-	-	
**14:0 iso**	1.18 ± 0.03	0.78 ± 0.01	0.01	1.985 ± 0.02	1.455 ± 0.04	0.007
**14:0**	0.215 ± 0.02	0.195 ± 0.01		1.07 ± 0.08	0.765 ± 0.011	
**15:1 iso G**	0.165 ± 0.01	0.15 ± 0.00		0.325 ± 0.02	-	
**15:0 iso**	5.75 ± 0.11	5.185 ± 0.06		14.29 ± 0.42	12.97 ± 0.20	
**15:0anteiso**	43.96 ± 0.72	46.15 ± 0.04		31.025 ± 0.93	33.495 ± 0.40	
**15:0**	-	-		0.795 ± 0.05	0.31 ± 0.01	0.03
**16:0 iso**	3.05 ± 0.09	1.965 ± 0.06	0.009	1.17 ± 0.01	1.11 ± 0.01	
**16:0**	1.75 ± 0.10	1.81 ± 0.00		3.94 ± 0.16	3.98 ± 0.06	
**15:0 2OH**	-	-		0.23 ± 0.01	0.205 ± 0.01	
**17:0 iso**	3.93 ± 0.07	3.55 ± 0.06		3.835 ± 0.01	3.695 ± 0.05	
**17:0 anteiso**	17.2 ± 0.44	18.24 ± 0.16		4.82 ± 0.17	5.57 ± 0.08	0.06
**17:0**	0.215 ± 0.04	0.2 ± 0.01		2.075 ± 0.011	0.96 ± 0.04	0.02
**18:0 iso**	1.555 ± 0.01	1.02 ± 0.01	0.0025	0.395 ± 0.04	0.375 ± 0.04	
**18:1w9c**	2.775 ± 0.04	3 ± 0.13		5.91 ± 0.03	6.13 ± 0.08	
**18:1w7C**	0.525 ± 0.01	0.58 ± 0.01		1.35 ± 0.03	1.515 ± 0.04	
**18:0**	6.75±0.014	6.93 ± 0.08		10.4 ± 0.17	10.58 ± 0.00	
**19:0 iso**	1.335±0.01	1.185 ± 0.01		1.07 ± 0.01	1.135 ± 0.04	
**19:0 anteiso**	3.61 ± 0.04	3.68 ± 0.03		0.97 ± 0.04	1.225 ± 0.02	0.04
**19:0**	0.245±0.04	0.235 ± 0.01		2.25 ± 0.18	1.145 ± 0.06	
**20:4 w6,9,12,15c**	0.315± 0.01	0.415 ± 0.01	0.005	0.42 ± 0.01	0.49 ± 0.01	0.04
**20:1 w9c**	2.49	-		4.835 ± 0.04	5.165 ± 0.04	
**20:0**	2.5 ± 0.056	2.615 ± 0.063		3.495 ± 0.08	4.055 ± 0.06	0.02
**20:0 iso**	0.375 ± 0.01	0.31 ± 0.014		-	-	

* *p*-value = comparison between aerobic condition; ** *p*-value = comparison between anaerobic condition.
